# The VEGF decoy receptor soluble Fms-like tyrosine kinase 1 binds to macrophages

**DOI:** 10.1007/s10456-025-09980-w

**Published:** 2025-05-02

**Authors:** Cleo C. L. van Aanhold, Qing Yong, Lisa Landman, Samiksha Sardana, Anouk B. Bouwmeester, Kyra L. Dijkstra, Ron Wolterbeek, Hailiang Mei, Rayman T. N. Tjokrodirijo, Arnoud H. de Ru, Peter A. van Veelen, Jan A. Bruijn, Cees van Kooten, Hans J. Baelde

**Affiliations:** 1 Department of Pathology, Leiden University Medical Center, Leiden, The Netherlands; 2 Biomolecular Mass Spectrometry and Proteomics, Bijvoet Center for Biomolecular Research and Utrecht Institute for Pharmaceutical Sciences, University of Utrecht, Utrecht, The Netherlands; 3 Netherlands Proteomics Center, Utrecht, The Netherlands; 4 Department of Pathology, Radboud University Medical Center, Nijmegen, The Netherlands; 5 Sequencing Analysis Support Core, Leiden University Medical Center, Leiden, The Netherlands; 6 Center for Proteomics and Metabolomics, Leiden University Medical Center, Leiden, The Netherlands; 7 Department of Nephrology, Leiden University Medical Center, Leiden, The Netherlands

**Keywords:** Soluble Fms-like Tyrosine kinase-1, Macrophage, Anti-angiogenesis, Inflammation

## Abstract

**Background:**

Soluble Fms-like Tyrosine kinase-1 (sFLT1) is a native inhibitor of VEGF, best known for its antiangiogenic effects in preeclampsia. sFLT1 also reduces chronic inflammation and promotes tissue repair. In experimental diabetic nephropathy, we previously found that sFLT1 ameliorates kidney fibrosis and reduces the infiltration of macrophages. How sFLT1 regulates inflammation is still incompletely understood. Based on the direct association of sFLT1 with various cell types, we here studied whether sFLT1 interacts with macrophages to modulate inflammation.

**Methods:**

Using various macrophage cell lines, sFLT1 cell surface binding was detected with flow cytometry. Enzyme studies, mass spectrometry and RNAseq were employed to identify potential sFLT1 cell surface interactors and effects of sFLT1 on macrophage signaling.

**Results:**

Soluble FLT1 binds to primary macrophages, THP-1 and RAW264.7 macrophages in vitro. Alternative activation with IL-4 increases sFLT1 binding in THP-1 macrophages, whereas proinflammatory activation with IFN-γ and LPS decreases binding. Binding of sFLT1 depends on heparan sulphates, and colocalizes with the membrane heparin sulfate proteoglycan neuropilin-1. Incubation with sFLT1 reduces the gene expression of chemokine receptors.

**Conclusion:**

Our results show that sFLT1, while typically associated with angiogenesis, also directly interacts with macrophages. Alternative activation of macrophages by IL-4 strongly increases binding of sFLT1 to the cell surface membrane, possibly via the VEGF co-receptor neuropilin-1. Considering sFLT1’s anti-inflammatory effects in animal studies, our findings indicate a novel function for sFLT1 to directly control anti-inflammatory macrophage function.

**Supplementary Information:**

The online version contains supplementary material available at 10.1007/s10456-025-09980-w.

## Introduction

Soluble Fms-like tyrosine kinase 1 (sFLT1) is an endogenous inhibitor of vascular endothelial growth factor (VEGF). By acting as a VEGF decoy receptor, sFLT1 determines the amount of VEGF available for signaling both systemically and in the tissue environment [1, 2]. In women with preeclampsia, high sFLT1 levels cause a systemic VEGF deficiency, and are associated with the development of glomerular dysfunction and endotheliosis [2, 3]. Recently, sFLT1 and its nephrotoxic effects have been suggested to play a role in other kidney diseases as well [4]. 

In addition to these well-known effects, there is growing evidence that sFLT1 also has anti-inflammatory properties. Treatment with sFLT1 markedly reduces chronic inflammation and promotes tissue repair in several inflammatory models such as atopic dermatitis, atherosclerosis, diabetic nephropathy, sepsis and arthritis [5–9]. Specifically, in experimental models of diabetic nephropathy and chronic dermatitis, we found that sFLT1 treatment lowered the tissue infiltration of macrophages and reversed the chronic tissue damage [5, 8]. Similarly, others have shown that sFLT1-knock out mice exhibit increased proinflammatory macrophage activation and a failure to resolve inflammation in heart failure [7]. Together these studies suggest that sFLT1 has therapeutic potential in chronic inflammatory conditions. 

The mechanisms whereby sFLT1 reduces chronic inflammation are not fully understood. Potential mechanisms that have been described rely on inhibiting the proinflammatory effects of VEGF and PLGF, including increased vascular permeability, induced expression of endothelial adhesion molecules and chemokines, and chemoattraction of FLT1-positive myeloid cells [7, 8, 10–13]. Interestingly, sFLT1 has also been demonstrated to directly interact with various cell types (podocytes, pericytes, endothelial cells, tumor cell lines) and control cell function independent of VEGF. Therefore, sFLT1 may also inhibit inflammation via a VEGF-independent pathway. In fibrotic kidneys, staining of the FLT1 ectodomain was localized in macrophages [14]. Hence, the anti-inflammatory effects of sFLT1 may perhaps also be explained by a direct interaction with macrophages.

In this study, we analyzed the potential binding of sFLT1 to the cell surface of macrophages. In addition, we aimed to identify sFLT1’s potential binding targets and effects on gene expression in macrophages to support our prior in vivo findings.

## Results

### sFLT1 binds to the cell surface of macrophages

To study whether sFLT1 interacts with macrophages, cells were incubated with recombinant human sFLT1, after which sFLT1 staining was measured using flow cytometry.

**Fig. 1:**
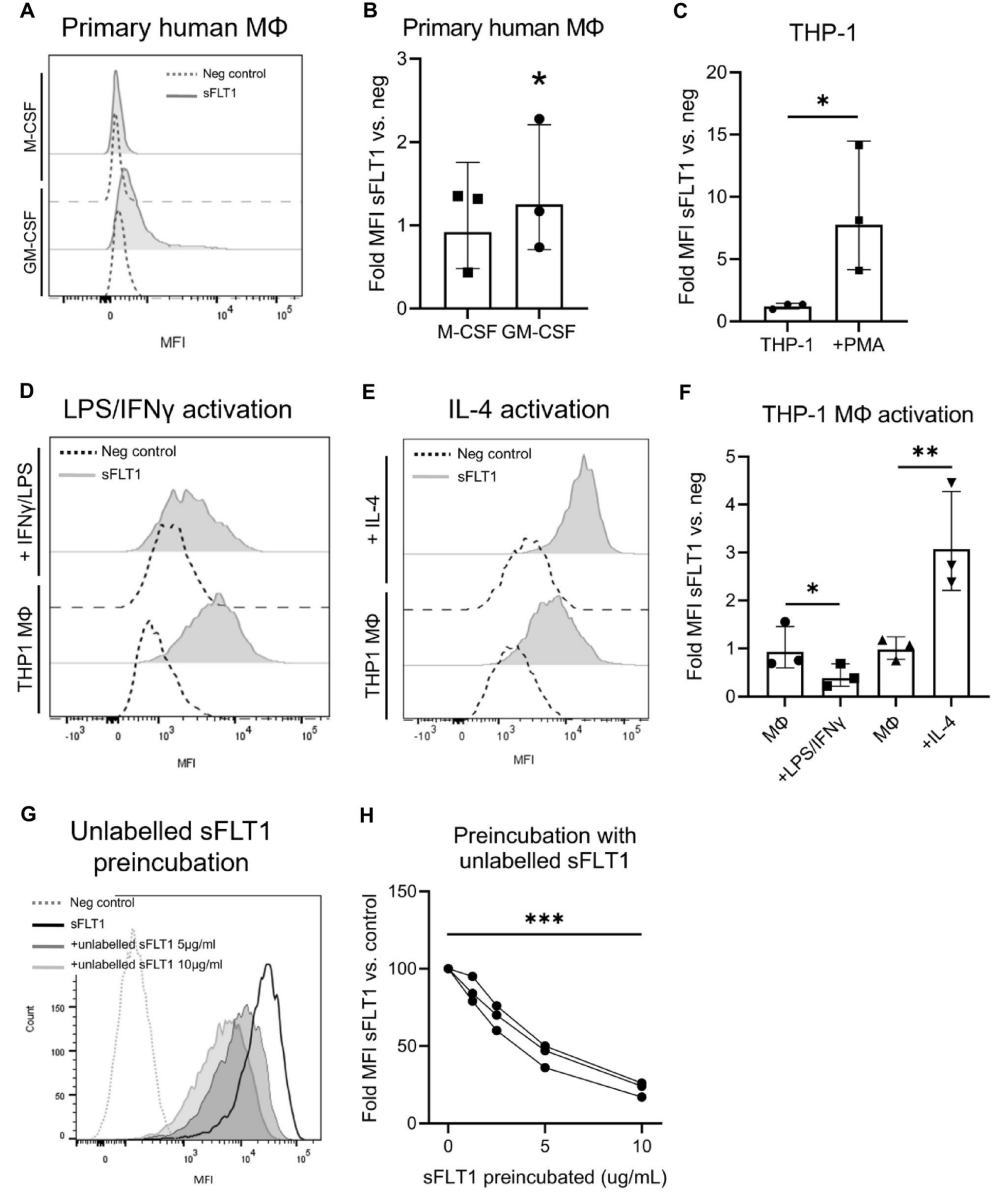
sFLT1 binds macrophage cell lines. **a, b** Primary monocytes were differentiated toward macrophages with GM-CSF or M-CSF, then sFLT1 binding was determined via indirect staining using flow cytometry. **a** Representative flow cytometry analysis. **b** Quantification of the flow cytometry data shows that sFLT1 binds MDMs differentiated with GM-CSF but not M-CSF. The summary data are presented as the geometric mean fold change in MFI (sFLT1/BSA) ± SD of n = 3 donors. *p < 0.05 refers to the difference in log MFI between sFLT1 and BSA-incubated GM-CSF-differentiated cells (linear mixed model, using the random effect of the individual donor). **c** sFLT1 binding determined in THP-1 monocytes and THP-1 macrophages (differentiated using PMA). Quantification of the flow cytometry data shows that sFLT1 binds THP-1 macrophages, but not THP-1 monocytes. The summary data are presented as the geometric mean fold change in MFI (sFLT1/BSA) ± SD of n = 3 independent experiments. *p < 0.05 refers to the interaction effect of sFLT1*PMA on log MFI (two-way ANOVA).** d**–**f** THP-1 macrophages were activated using IFN-γ + LPS or IL-4, then sFLT1 binding was determined. **d, e** Representative flow cytometry analysis. (F) Quantification of the flow cytometry data shows that sFLT1 binding is decreased by IFN-γ + LPS and increased by IL-4. The summary data are presented as the geometric mean fold change in MFI (sFLT1/BSA) ± SD of n = 3 independent experiments. *p < 0.05 and **p < 0.01 refer to the interaction effect sFLT1*activation on log MFI (two-way ANOVA). **g, h** IL-4-activated THP-1 macrophages were preincubated with unlabelled sFLT1, and then sFLT1 binding was measured. **g** Representative flow cytometry analysis. **h** Quantification of flow cytometry data shows that preincubation with unlabelled sFLT1 blocks sFLT1 membrane association. The data represent the geometric mean fold change in MFI (sFLT1/neg control) ± SD of 3 independent experiments. ***p < 0.001 refers to the effect of log unlabelled sFLT1 concentration on log MFI (linear regression)

For primary human monocyte-derived-macrophages (MDMs), we found that sFLT1 bound to these macrophages when differentiated with GM-CSF, but not M-CSF, compared to the negative control conditions (Fig. 1a, b). In line with our findings using human macrophages, we discovered that similarly murine sFLT1-VSV-tagged protein bound to murine RAW264.7 macrophages (data not shown).

In a next step we studied sFLT1 binding to THP-1 cells. THP-1 monocytes only showed minimal sFLT1 binding compared to negative control cells. In contrast, THP-1 cells which were differentiated toward macrophages using PMA (called “THP-1 macrophages” hereafter) showed marked cell surface binding of sFLT1 (Fig. 1c). Thus, macrophage cell surface binding of sFLT1 is a conserved property across various macrophage cell lines.

### Activation with IFN-γ/LPS reduces sFLT1 binding, while activation with IL-4 increases binding of sFLT1 to the macrophage cell surface

We further explored the effect of two different activation states on the binding of sFLT1 to THP-1 macrophages. First, THP-1 macrophages were activated towards a proinflammatory phenotype using IFN-γ and LPS, leading to a marked upregulation of TNF-α, IL-6 and iNOS at the mRNA level (Supplementary Table 1). After incubation with IFN-γ and LPS, sFLT1 binding to the cell surface was decreased as compared to non-activated macrophages (Fig. 1d, f).

Furthermore, THP-1 macrophages were activated towards an alternatively activated phenotype using IL-4, characterized by upregulation of IL-10 and MRC1 mRNA expression (Supplemental Table 1). In contrast to the finding in IFN-γ + LPS macrophages, sFLT1 binding to the cell surface of IL-4-activated macrophages was significantly increased compared to binding to non-activated macrophages (Fig. 1e, f). Thus, different activation states in macrophages are associated with different binding capacities of sFLT1.

**Table 1:** RNA sequencing reveals differentially expressed genes following sFLT1 incubation in IL-4-activated THP-1 macrophages

	Gene	log_2_ CPM	Log_2_ Fold change	FDR	Full gene name
Stimulation	DGCR5	2.906	0.963	0.034	DiGeorge syndrome critical region gene 5
	RASGRP3	4.267	0.925	0.000	RAS guanyl releasing protein 3
	SLC5A3	5.908	0.834	0.000	solute carrier family 5 member 3
	SLCO4C1	4.852	0.724	0.025	solute carrier organic anion transporter family member 4C1
	LINC02541	4.003	0.710	0.012	long intergenic non-protein coding RNA 2541
	SAMD4A	4.671	0.705	0.007	sterile alpha motif domain containing 4A
	CCL2	4.926	0.660	0.049	C–C motif chemokine ligand 2
	RAB42	4.935	0.616	0.015	RAB42, member RAS oncogene family
	SLAMF7	5.761	0.612	0.001	SLAM family member 7
	DENND2D	5.080	0.574	0.033	DENN domain containing 2D
	ATP6V1C2	5.401	0.567	0.033	ATPase H + transporting V1 subunit C2
	MTATP6P1	5.588	0.561	0.049	MT-ATP6 pseudogene 1
	RND3	6.916	0.545	0.012	Rho family GTPase 3
Reduction	PCDH12	2.830	− 1.022	0.012	protocadherin 12
	SLC16A10	3.349	− 0.860	0.049	solute carrier family 16 member 10
	ARRDC3	4.047	− 0.832	0.000	arrestin domain containing 3
	CX3CR1	5.136	− 0.763	0.000	C-X3-C motif chemokine receptor 1
	CCR2	3.571	− 0.725	0.033	C–C motif chemokine receptor 2
	ODC1	6.199	− 0.663	0.000	ornithine decarboxylase 1
	MYBL2	7.097	− 0.481	0.012	MYB proto-oncogene like 2
	CD93	7.924	− 0.393	0.049	CD93 molecule
	HMGN2	8.480	− 0.379	0.049	High mobility group nucleosomal binding domain 2

FDR, Benjamini and Hochberg false discovery rate

To further confirm the specificity of sFLT1 interaction to IL-4-activated macrophages, we preincubated these cells with increasing concentrations of unlabelled sFLT1, which lowered the interaction of sFLT1 in a dose-dependent manner (Fig. 1g, h).

### Cellular uptake of sFLT1 by macrophages

**Fig. 2:**
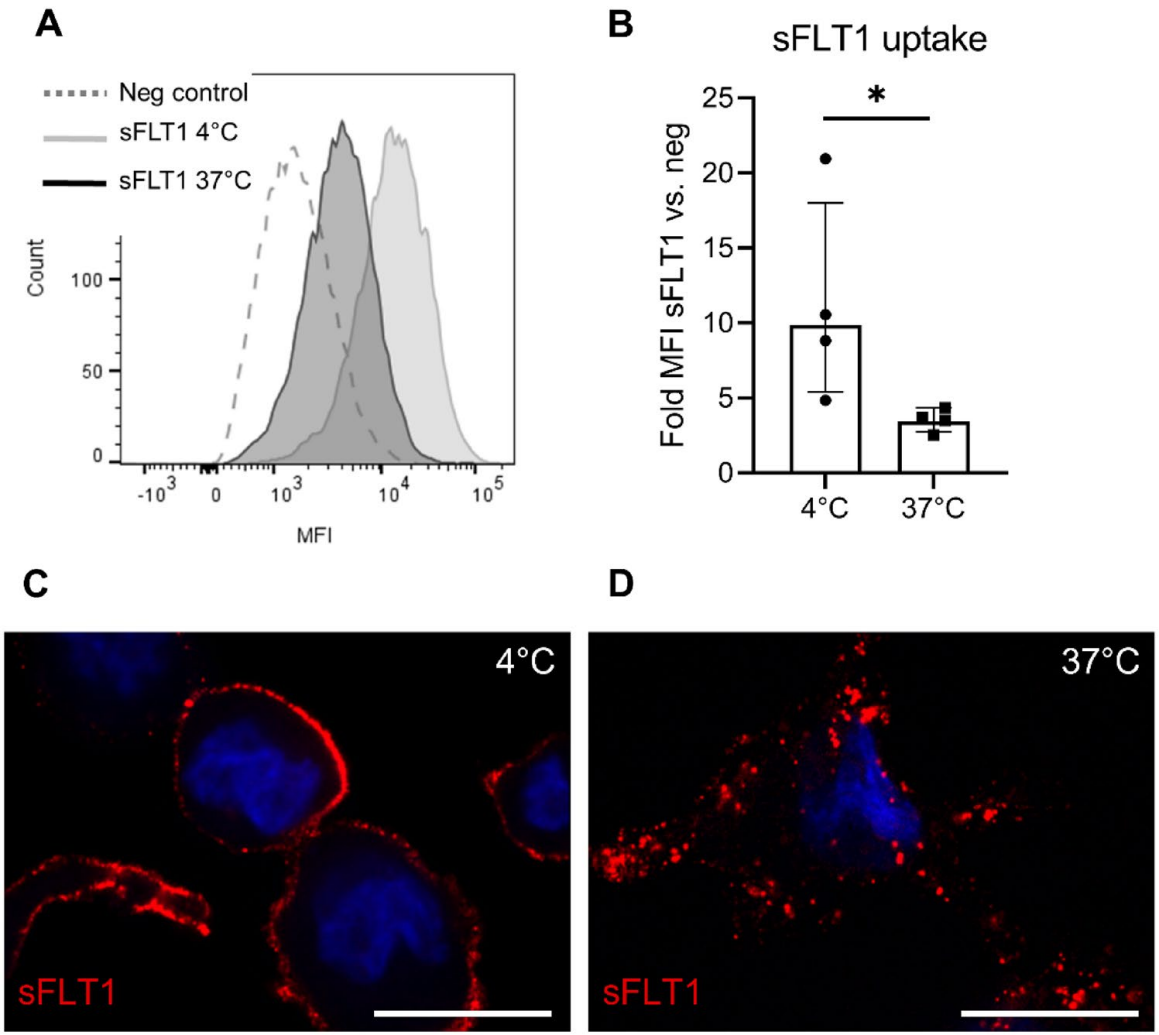
Cellular uptake of sFLT1 in THP-1 macrophages. Following binding of sFLT1 (30 min, 4 °C) to IL-4-activated THP-1 macrophages, cells were further incubated at 4 or 37 °C for 30 min. Then membrane sFLT1 staining was measured using flow cytometry. **a** Representative flow cytometry analysis. **b** Quantification of the flow cytometry data shows that membrane sFLT1 is reduced following incubation at 37 °C, as compared to controls incubated at 4 °C. The summary data are presented as the geometric mean fold change in MFI (sFLT1/BSA) ± SD of n = 3 independent experiments. *p < 0.05, refers to the interaction effect sFLT1*temperature on log MFI (two-way ANOVA). Representative images of sFLT1-treated THP-1 macrophages show **c** cell surface staining of sFLT1 at 4 °C and **d** a granular staining pattern of sFLT1 at 37 °C, indicative of cellular uptake of sFLT1. Scale bar, 20 μm

Next, we studied the potential uptake of bound sFLT1 by THP-1 macrophages. Since IL-4-activated macrophages showed the highest level of sFLT1 binding, these cells were used for subsequent experiments. Cells were incubated with sFLT1 for 30 min at 4 °C, after which the cells were washed to remove unbound sFLT1. To observe protein uptake, cells were further incubated for 30 min at either 4 or 37 °C, followed by staining for sFLT1. While cells incubated at 4 °C showed marked surface binding of sFLT1, cells incubated at 37 °C showed a decreased detection of surface sFLT1 (Fig. 2a, b). To determine whether this effect was due to cellular uptake of sFLT1, the cells were permeabilized before staining. Indeed, confocal microscopy revealed a granular staining pattern of sFLT1 in cells incubated at 37 °C (Fig. 2c, d). Together these findings indicate that sFLT1 is internalized by THP-1 macrophages.

### sFLT1 regulates chemokine receptor expression in IL-4-activated macrophages

**Fig. 3:**
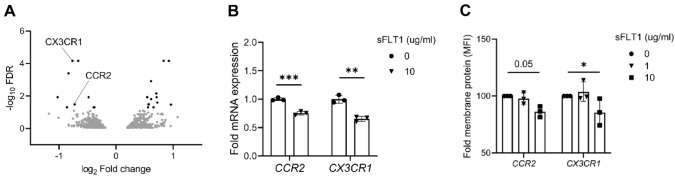
sFLT1 alters gene expression in IL-4-activated THP-1 macrophages. **a** Volcano plot showing differentially expressed genes following sFLT1 binding in IL-4-activated THP-1 macrophages (genes with FDR < 0.05, black). **b** *CCR2* and *CX3CR1* mRNA levels were measured in IL-4-activated macrophages incubated with BSA (dots) or sFLT1 (triangle). mRNA expression levels are displayed relative to control cells. n = 3 experiments. **p < 0.01 and ***p < 0.001, refers to effect of sFLT1 incubation on log mRNA expression of indicated gene (t-test). **c** CCR2 and CX3CR1 membrane protein levels were measured in IL-4-activated macrophages after stimulation with sFLT1 (24 h). Fold MFI is displayed relative to control cells. n = 3 experiments. *p < 0.05, refers to the effect of log sFLT1 concentration on log MFI (linear mixed model, using the random intercept of experiment)

To determine the potential effects of sFLT1 on the gene expression, IL-4-activated THP-1 macrophages were incubated with BSA or sFLT1 for 24 h. Hereafter, RNA was isolated and sequenced to look for transcriptional changes. sFLT1 incubation elicited the differential expression of n = 22 genes (FDR < 0.05, Table 1), including the chemokine receptors genes *CX3CR1* and *CCR2*, suggestive of a downregulatory effect on cell migration. Using qPCR and flow cytometry, we further studied several top differentially expressed genes. In line with the RNA-sequencing results, sFLT1 incubation differentially regulated the mRNA and protein expression of the indicated genes (Fig. 3).

### Mass spectrometry analysis for identification of binding partners for sFLT1

Our data show specific binding of sFLT1 to the macrophage cell surface, especially to IL-4-activated macrophages, resulting in a downregulation of chemokine receptors. We hypothesized that this binding is mediated by potential interactors that might have been upregulated at the mRNA level following IL-4 activation. To identify potential binding partners of sFLT1 at the IL-4-activated macrophage cell surface, cell surface-bound sFLT1 and interacting proteins were purified from the cell lysates and analyzed using mass spectrometry. Upregulated proteins were ranked based on abundance ratio, filtered by cellular location (cell surface and extracellular space, Gene ontology) (Table 2). Next, we utilized RNA sequencing to identify potential IL-4-induced binding targets in our THP-1 macrophages. This yielded a selection of eight proteins MYH9, CAP1, NCKAP1L, SLC30A1, IQGAP1, ANXA2, TLN1, SIPA1L3, which were identified with both mass spectrometry and RNA sequencing. The potential interactions between these eight proteins and sFLT1 were then further analyzed using Alphafold2 prediction models, however, we were unable to confirm any direct interactions (data not shown). Interestingly, interaction with sFLT1 has been documented before for two of the top hits identified with mass spectrometry, the heparan sulphate proteoglycans neuropilin-1 (NRP1) and perlecan (HSPG2) [15, 16]. Therefore, these were chosen for further study.

**Table 2:** List of top proteins (defined by abundance ratio) identified with mass spectrometry

Protein	Gene name	Abundance ratio (sFlt1/PBS)	p-value	Coverage	IL-4-upregulated	Notes
Myosin-9	MYH9	4.06	0.00	49	Yes	
Neuropilin-1	NRP1	3.78	0.00	27		Heparan sulphate proteoglycan
Ras-related C3 botulinum toxin substrate 1	RAC1	3.29	0.16	40		
ADP-ribosylation factor 3	ARF3	3.21	0.01	66		
Catechol O-methyltransferase	COMT	3.09	0.03	53		
Adenylyl cyclase-associated protein 1	CAP1	3.02	0.01	71	Yes	
Vimentin	VIM	2.81	0.04	72	Yes	
Alpha-actinin-1	ACTN1	2.56	0.29	35		
Heat shock protein HSP 90-beta	HSP90AB1	2.50	0.04	49		
Endoplasmin	HSP90B1	2.38	0.14	47		
Heat shock protein HSP 90-alpha	HSP90AA1	2.00	0.19	50		
X-ray repair cross-complementing protein 5	XRCC5	1.99	0.40	30		
Nck-associated protein 1-like	NCKAP1L	1.93	0.23	55	Yes	
Protein disulfide-isomerase A6	PDIA6	1.91	0.45	39		
WD repeat-containing protein 1	WDR1	1.80	0.53	62	Yes	
Chloride intracellular channel protein 1	CLIC1	1.78	0.67	56		
60 kDa heat shock protein, mitochondrial	HSPD1	1.75	0.37	67		
Endoplasmic reticulum chaperone BiP	HSPA5	1.70	0.42	62		
Catalase	CAT	1.68	0.44	69	Yes	
Ras GTPase-activating-like protein IQGAP1	IQGAP1	1.65	0.48	49	Yes	
Elongation factor 2	EEF2	1.55	0.58	50		
Calreticulin	CALR	1.52	0.85	45		
Integrin beta-2	ITGB2	1.51	0.62	52		
Cell division control protein 42 homolog	CDC42	1.49	0.76	63	Yes	
Fatty acid synthase	FASN	1.48	0.82	31		
Annexin A2	ANXA2	1.47	0.66	75	Yes	
Alpha-2-macroglobulin receptor-associated protein	LRPAP1	1.45	0.70	61		
Elongation factor 1-alpha 1	EEF1A1	1.35	0.83	66		
ATP-dependent RNA helicase DDX3X	DDX3X	1.32	0.85	50		
Amiloride-sensitive amine oxidase [copper-containing]	AOC1	1.30	0.87	44		
Heat shock cognate 71 kDa protein	HSPA8	1.28	0.90	70		
Talin-1	TLN1	1.22	0.94	47	Yes	
Signal-induced proliferation-associated 1-like protein 3	SIPA1L3	1.17	0.96	50	Yes	
Vascular endothelial growth factor receptor 1	FLT1	1.13	0.98	32		Bait
Basement membrane-specific heparan sulfate proteoglycan core protein	HSPG2	0.66	0.31	52	Yes	Heparan sulphate proteoglycan

Proteins were filtered by cell surface location and coverage (> 25%). The bait protein (sFLT1) and a selection of candidate membrane-associated proteins are shown here

### sFLT1 binding to macrophages depends on heparan sulphate binding

**Fig. 4:**
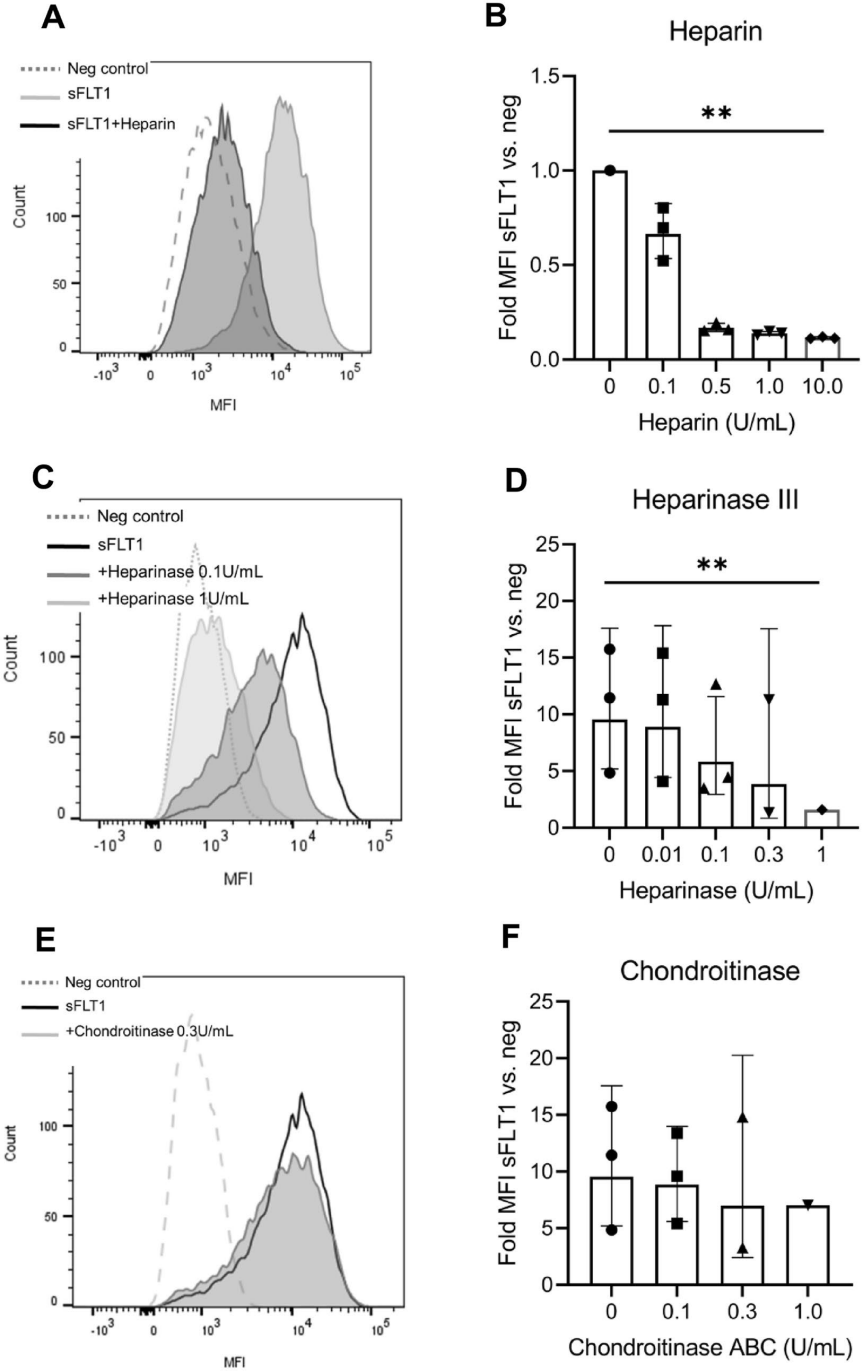
sFLT1-macrophage cell surface association depends on heparan sulfate binding. IL-4-activated THP-1 macrophages were co-incubated with sFLT1 and heparin. Then sFLT1 binding was determined using flow cytometry. **a** Representative flow cytometry analysis. **b** Quantification of the flow cytometry data shows that coincubation with heparin blocks sFLT1-membrane association. The data represent the geometric mean fold change in MFI (sFLT1/BSA) ± SD of 3 independent experiments. **p < 0.01, refers to the effect of log heparin concentration on log MFI (linear regression). IL-4-activated THP-1 cells were pre-treated with heparinase III, and then incubated with sFLT1. **c** Representative flow cytometry analysis. **d** Quantification of the flow cytometry data shows that preincubation with heparinase III reduces sFLT1 association. The data represent the geometric mean fold change in MFI (sFLT1/BSA) ± SD of 3 independent experiments. **p < 0.01, refers to the interaction effect of sFLT1*heparinase on log MFI (linear mixed model, using the random effect of experiment). IL-4-activated THP-1 were pre-treated with chondroitinase ABC, and then incubated with sFLT1. **e** Representative flow cytometry analysis. **f** Quantification of the flow cytometry data shows that preincubation with chondroitinase does not affect sFLT1 binding. The data represent the geometric mean fold change in MFI (sFLT1/BSA) ± SD of 3 independent experiments

Since we identified the upregulation of glycosaminoglycans neuropilin-1 and perlecan in our mass spectrometry analysis, we further explored whether sFLT1 binds to GAGs at the macrophage cell surface. First, we found that co-incubation of sFLT1 with heparin dose-dependently reduced the binding of sFLT1 to macrophages (Fig. 4a, b). In line with this, pre-incubation of cells with heparinase III abolished the subsequent binding of sFLT1 to the macrophage cell surface (Fig. 4c, d). This inhibition was not observed upon treatment with chondroitinase ABC (Fig. 4e, f). Thus, binding of sFLT1 to the macrophage cell surface depends on heparan sulfate interaction.

### sFLT1 binding may associate with neuropilin-1 at the IL-4-macrophage cell surface

**Fig. 5:**
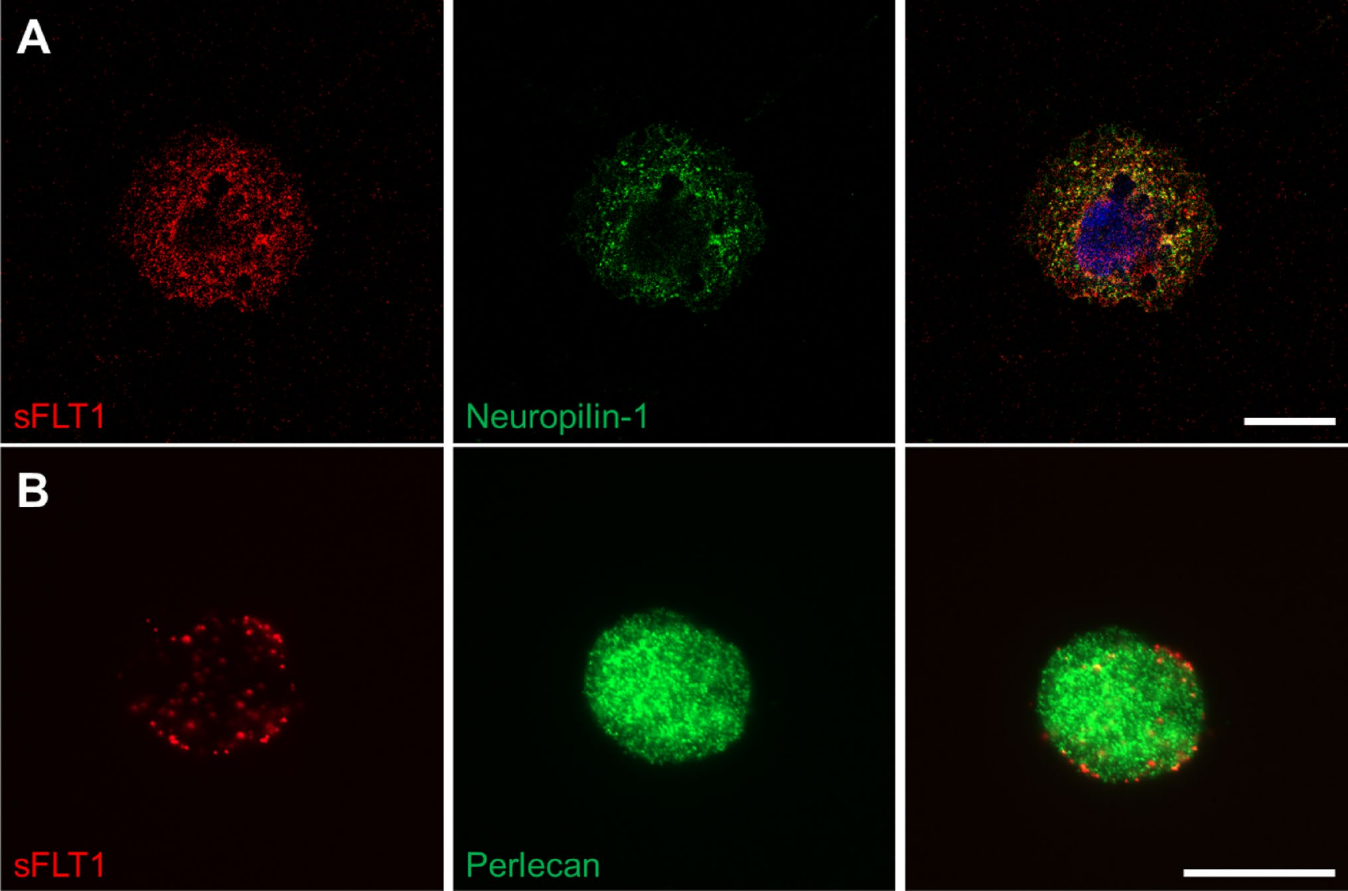
sFLT1 colocalizes with cell surface neuropilin-1. IL-4-activated THP-1 macrophages were incubated with Alexa Fluor-647-tagged sFLT1. Then cells were stained with anti-neuropilin-1 and anti-perlecan antibodies. Double-label immunostaining of sFLT1 and neuropilin-1 revealed **a** colocalization of sFLT1 with neuropilin-1 at the macrophage cell surface. **b** Double-label immunostaining of sFLT1 and perlecan shows minimal colocalization, suggesting these proteins do not interact at the cell surface. Scale bars 20 µm

Lastly, we went on to study the direct interaction of sFLT1 with perlecan and neuropilin-1 in IL-4 activated THP-1 macrophages. In our RNAseq data (THP-1 macrophages BSA vs. IL-4), we found that perlecan (2.5-fold), but not neuropilin-1, was upregulated by IL-4. This was confirmed by qPCR. Double-label immunostaining revealed marked colocalization of sFLT1 with neuropilin-1, but not perlecan in THP-1 macrophages, suggesting that sFLT1 binds in complex with neuropilin-1 at the macrophage cell surface (Fig. 5).

## Discussion

**Fig. 6:**
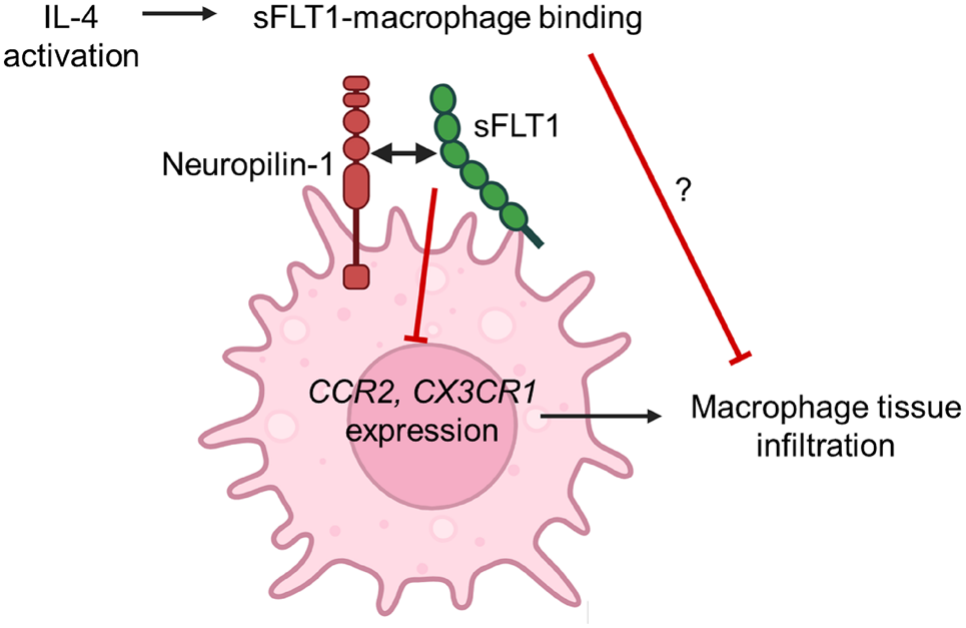
sFLT1 binds to macrophages. sFLT1 binds to IL-4-activated macrophages via neuropilin-1 heparan sulphate. In macrophages, sFLT1 binding leads to a downregulation of chemokine receptors CCR2 and CX3CR1, thereby reducing tissue infiltration of macrophages and inflammation in vivo

We set out to investigate a potential direct interaction between sFLT1 and macrophages. While the adverse effects of high sFLT1 titers are well-known, the function of sFLT1 in normal physiology is incompletely understood. Previously, we found that treatment with sFLT1 reverses chronic inflammation and promotes tissue repair. Based on early studies demonstrating direct interactions of sFLT1 with podocytes and endothelial cells, we here studied the potential interaction of sFLT1 with macrophages. Our results indicate that sFLT1 binds to macrophages using various macrophage cell lines in vitro. Interestingly, activation of macrophages toward an anti-inflammatory phenotype with IL-4 increases their capacity to bind sFLT1, while proinflammatory activation with IFN-γ and LPS decreases sFLT1 binding. sFLT1-macrophage binding depends on heparan sulphate interaction, possibly in complex with the heparan sulphate proteoglycan neuropilin-1 (Fig. 6). Thus, we present direct interaction of sFLT1 with macrophages, suggesting a new anti-inflammatory mechanism.

Direct interactions of sFLT1 with different cell types have been documented before. In endothelial cells, sFLT1 adherence to cell surface α5β1 integrin and neuropilin-1 promotes endothelial cell motility, angiogenesis and monocyte adhesion [17–19]. In podocytes and pericytes, interaction of sFLT1 with the GM3 ganglioside maintains cell morphology and adhesion [20]. In the current study, we identified sFLT1 association to several macrophage cell lines. This interaction was abrogated by heparinase treatment, suggesting that sFLT1’s heparin-binding fourth Ig-domain is the epitope for macrophage binding. This is similar to sFLT1’s interaction to the ganglioside GM3 at the podocyte cell surface, also mediated by its fourth Ig-domain [20]. Considering the marked colocalization of sFLT1 and neuropilin-1 at the macrophage cell surface, combined with the identification of neuropilin-1 as a potential sFLT1 interactor using mass spectrometry, we infer that sFLT1 binds the macrophage cell surface in complex with neuropilin-1. Two sFLT1 epitopes have been reported to bind the neuropilin-1 ectodomain, the second Ig-like domain and the fourth heparin-binding domain of sFLT1, thereby blocking neuropilin-1 binding to VEGF [15, 18]. While IL-4 activation of macrophages led to an increase in sFLT1 binding, IL-4 did not upregulate neuropilin-1 mRNA expression (data not shown). This may indicate that IL-4 promotes the bioavailability of cell surface neuropilin-1 in another way, possibly by structural reorganization or by increased localization to the plasma membrane, potentially induced by post-translational modifications.

How does this novel concept of direct sFLT1-macrophage interaction fit in with the described anti-inflammatory effects of sFLT1? In models of diabetic nephropathy, heart fibrosis, and atopic dermatitis, treatment with sFLT1 effectively reduced chronic inflammation and promoted tissue repair [5, 7, 8]. In these studies, treatment with sFLT1 led to a reduction in the tissue infiltration of macrophages as well as to their polarization towards an anti-inflammatory phenotype. Considering our observation that anti-inflammatory macrophages strongly bind sFLT1, it is possible that sFLT1 mediates anti-inflammatory effects by promoting the function of anti-inflammatory macrophages, for example by altering signaling or by promoting their tissue positioning. Although sFLT1 downregulated the expression of chemokine receptors in macrophages, we did not identify effects of sFLT1 on the activation state of macrophages. Reasons for this may be the lack of a cofactor in our experiments (e.g. VEGF), or the long duration of sFLT1 incubation as compared to incubation experiments in podocytes [20]. Alternative to potential signaling effects, it is also possible that sFLT1 binds anti-inflammatory macrophages to regulate their tissue positioning. As seen in Fig. 3, sFLT1 lowers both the mRNA and protein levels of chemokine receptors that are essential for cell migration. Neuropilin-1-expressing macrophages identify an anti-inflammatory macrophage population that promotes the resolution of inflammation and tissue repair [21–24]. Hence, it is possible that sFLT1 mediates the recruitment of neuropilin-1-positive macrophages towards sites of tissue injury, thereby promoting the resolution of inflammation and tissue repair.

The concept that sFLT1 has direct immunomodulating properties is relevant to the pathogenesis of preeclampsia. In preeclampsia, placental production of sFLT1 leads to high systemic sFLT1 levels. High sFLT1 titers induce glomerular endotheliosis, proteinuria and hypertension in preeclampsia. Still, the purpose of fetal sFLT1 production in pregnancy is poorly understood. Although speculative, one report suggested that sFLT1 may represent a fetal escape mechanism that modulates an excessive proangiogenic response of decidua [25]. Alternatively, considering our present observations of direct sFLT1-macrophage interaction, it is also intriguing to speculate that fetal sFLT1 functions to control the maternal inflammatory response seen in preeclampsia [26].

In conclusion, our results support a novel concept whereby the antiangiogenic protein sFLT1 has an immunomodulating effect on macrophages. This novel mechanism supports animal studies [5, 7, 8], in which sFLT1 was shown to have powerful anti-inflammatory effects in models of chronic inflammation, including experimental diabetic nephropathy. Future research should further study the precise consequences of sFLT1 signaling in inflammation-related diseases.

## Methods

### Cell culture

Human monocytic THP-1 cells were maintained in RPMI 1640 culture medium (10% FCS, 1% P/S). THP-1 cells were differentiated toward THP-1 macrophages by incubation with 10 ng/mL phorbol 12-myristate 13-acetate for 48 h (PMA, Sigma, P8139), followed by “resting incubation” in full media for 24 h. THP-1 macrophages were then activated toward proinflammatory macrophages by incubation with 20 ng/ml of IFN-γ (R&D) and 1 ng/ml LPS (Sigma) for 24 h. Anti-inflammatory macrophage activation was obtained by incubation with 10 ng/ml of IL-4 (Sino Biologicals) for 48 h. Mouse RAW264.7 cells were maintained in DMEM culture medium (10% FBS, 1% P/S). Primary monocytes obtained from a buffy coat were differentiated toward macrophages using RPMI 1640 media (10% FCS and 1% PS) containing either GM-CSF or M-CSF for 72 h, as was described previously [27].

### Flow cytometry

For detection of sFLT1 binding in macrophages, both indirect and direct detection methods were used in this project. For indirect detection of sFLT1 binding in human cell lines, cells were incubated with BSA or sFLT1-His protein (5 µg/ml, Sino Biological) and cell surface sFLT1 was detected using mouse anti-His (1:1000, MA1-21315, Invitrogen) and Alexa Fluor 647 anti-mouse (1:600, Thermo Fisher) antibodies. For indirect detection of sFLT1 binding in murine RAW264.7 macrophages, incubation with murine sFLT1-VSV protein and VSV as a control were used [8], which were detected using anti-VSV (1:9000, Abcam, 1.0 mg/mL, ab19257) and Alexa Fluor 647 anti-rabbit (1:600, Thermo Fisher) antibodies. In heparin and enzyme studies, a directly labelled sFLT1-His-AF647 protein was used (900 ng/ml).

Before sFLT1 incubation, cells were washed twice with PBA (1% BSA/PBS). Cells were then detached with 5 mM disodium EDTA, pH 7.2, at 37 °C for 5 min. 200,000 cells were incubated with sFLT1 at 4 °C for 30 min while shaking. Washing cells three times with PBA between every incubation step, cells were then consecutively incubated with primary and secondary antibodies for 30 min and finally stained with propidium iodide for 10 min. Analysis was performed using a BD FACSCanto-II 3L flow cytometer (BD Biosciences, San Jose, CA, USA). Cyto-Cal beads FC3MV (Distrilab, Leusden, Netherlands) were used for calibration allowing comparison of different measurements over time.

To study the specificity of sFLT1 binding, IL-4 activated THP-1 macrophages were first incubated with indicated concentrations of non-labeled sFLT1 protein, followed by washing and staining with labeled sFLT1 protein (900 ng/ml).

For internalization studies, in-between sFLT1 and primary antibody incubation steps, cells were incubated at 4 or 37 °C for 30 min. Cell surface sFLT1-His was indirectly detected using the above-mentioned protocol.

For heparin and enzyme experiments, cells were co-incubated with sFLT1 and heparin (LEO), or pretreated with heparinase III (37290-86-1, Sigma) or chrondroitinase ABC (9024-13-9, Sigma) prior to sFLT1 incubation.

For CCR2 and CX3CR1 protein measurement, IL-4 activated THP-1 macrophages were preincubated with sFLT1-His for 24 h, then a directly labelled CCR2-BV605 (1:40, Bio legend) or CX3CR1-AF647 (1:80, Bio legend) protein was used.

Figures show the fold differences in geometric mean fluorescence intensity (MFI) between sFLT1, CCR2 or CX3CR1incubated and negative control cells. Groups were compared using the statistical methods described below.

### Cellular staining

Cells were grown on glass coverslip slides in 24-well dishes and washed three times with PBS/1% BSA (PBA). Cells were incubated with sFLT1 as described in Flow cytometry methods section, and then stained with anti-neuropilin-1 (5 μg/ml 1 h, AF3870, R&D) and anti-perlecan (1:20, A7L6, GeneTex) antibodies. Cells were fixed with 4% buffered paraformaldehyde for 15 min and permeabilized with 100% ice-cold methanol for 15 min. Cells were further stained as described in Flow cytometry methods section. For mounting, EverBrite Mounting Medium with DAPI was used.

### Receptor identification

IL-4-activated THP-1 macrophages were dissociated and incubated with sFLT1-His (4C, 30 min). Then, cells were lysed using lysis buffer (50 mM Na2HPO4, 300 mM NaCl, 10 mM imidazole, protease inhibitors, 1% Triton X-100; pH 7.4; 30 min) and sonication (3 × 10 s). His-containing protein complexes were then purified from the cell lysates using HisPur Cobalt Resin beads, using manufacturer’s protocol (Thermo Fisher).

Samples were loaded onto a 4–12% Bis–Tris gradient gel (Invitrogen) and run for 1.5 cm. Subsequently, each lane was cut into 6 bands. Gel slices were first washed 3 × with water, and subsequently subjected to reduction with 10 mM dithiothreitol, alkylation with 50 mM of iodoacetamide, and in-gel trypsin digestion using a Proteineer DP digestion robot (Bruker). After addition of trypsin (at 12.5 ng/µl) and swelling of the bands, the bands were transferred to Eppendorf vials and the bands were covered in 25 mM NH4HCO3 pH 8.3. Tryptic digestion took place overnight at 37 C and the peptides were extracted from the gel slices with with 50/50/0.1 v/v/v water/acetonitril/formic acid. Finally peptides were lyophilized.

Tryptic peptides were dissolved in water/formic acid (100/0.1 v/v) and subsequently analyzed by on‐line C18 nanoHPLC MS/MS with a system consisting of an Ultimate3000nano gradient HPLC system (Thermo, Bremen, Germany), and an Exploris480 mass spectrometer (Thermo). Fractions were injected onto a cartridge precolumn (300 μm × 5 mm, C18 PepMap, 5 µm, 100 A, and eluted via a homemade analytical nano-HPLC column (50 cm × 75 μm; Reprosil-Pur C18-AQ 1.9 µm, 120 A (Dr. Maisch, Ammerbuch, Germany). The gradient was run from 2 to 40% solvent B (20/80/0.1 water/acetonitrile/formic acid (FA) v/v) in 40 min. The nano-HPLC column was drawn to a tip of ∼10 μm and acted as the electrospray needle of the MS source. The mass spectrometer was operated in data-dependent MS/MS mode for a cycle time of 3 s, with a HCD collision energy at 30 V and recording of the MS2 spectrum in the orbitrap, with a quadrupole isolation width of 1.2 Da. In the master scan (MS1) the resolution was 120,000, the scan range 400–1500, at standard AGC target @maximum fill time of 50 ms. A lock mass correction on the background ion m/z = 445.12 was used. Precursors were dynamically excluded after n = 1 with an exclusion duration of 10 s, and with a precursor range of 20 ppm. Charge states 2–5 were included. For MS2 the first mass was set to 110 Da, and the MS2 scan resolution was 30,000 at an AGC target of 100% @maximum fill time of 60 ms.

In a post-analysis process, raw data were first converted to peak lists using Proteome Discoverer version 2.2 (Thermo Electron), and submitted to the Uniprot database (Homo sapiens, 20,596 entries), using Mascot v. 2.2.07 (www.matrixscience.com) for protein identification. Mascot searches were with 10 ppm and 0.02 Da deviation for precursor and fragment mass, respectively, and trypsin as enzyme. Up to two missed cleavages were allowed. Methionine oxidation and acetyl on protein N-terminus were set as a variable modification; carbamidomethyl on Cys, were set as a fixed modification. Protein FDR was set to 1%. Normalization was on total peptide amount.

### RNA sequencing

RNA was extracted from 4 different treatment groups: differentiated THP-1 macrophages treated with BSA or IL-4 for 48 h, and from IL-4-activated macrophages further treated with BSA (10ug/ml) or sFLT1 (10ug/ml) for 24 h. RNA was isolated using TRIzol, RNA concentration was measured using nanodrop and quality and integrity of RNA were controlled with an Agilent 2100 Bioanalyzer (RIN values 9.6–10).

RNA-Seq FASTQ files were processed using the opensource BIOWDL RNAseq pipeline v5.0.0 developed at the LUMC. This pipeline performs FASTQ preprocessing (including quality control, quality trimming, and adapter clipping), RNA-Seq alignment, and read expression quantification FastQC was used for checking raw read QC. Adapter clipping was performed using Cutadapt (v2.10) with default settings. RNA-Seq reads’ alignment was performed using STAR (v2.7.5a) on GRCh38 human reference genome. The gene read quantification was performed using HTSeq-count (v0.12.4) with setting “–stranded = yes”. The gene annotation used for quantification was Ensembl version 104. Using the gene read count matrix, CPM was calculated per sample on all annotated genes. Genes with a CPM higher than 1 in at least 25% of all samples are kept for downstream analysis. For the differential gene expression analysis, dgeAnalysis R-shiny application was used. EdgeR (v3.28.1) with TMM normalization was used to perform differential gene expression analysis. Benjamini and Hochberg FDR was computed to adjust p-values obtained for each differentially expressed gene. Using a cutoff of 0.05 at the adjust p-values, we identified all up and down regulated genes.

### qPCR

For qPCR experiments, the primers listed in Table 3 were used.

**Table 3:** qPCR primers

Gene	Forward	Reverse
GAPDH	CGACCACTTTGTCAAGCTCA	AGGGGTCTACATGGCAACTG
CCR2	CCAGGACTGCCTGAGACAAG	TGGTGACTTCTTCACCGCTC
CXCR3C1	CAGAGGTTCCCTTGGCAGTC	GCCAATGGCAAAGATGACGG
IL-10	GTGATGCCCCAAGCTGAGA	CACGGCCTTGCTCTTGTTTT
TNF-alpha	CTGCTGCACTTTGGAGTGAT	AGATGATCTGACTGCCTGGG
IL-6	AGCCACTCACCTCTTCAGAAC	GCCTCTTTGCTGCTTTCACAC
IL-1b	GTGGCAATGAGGATGACTTGTTC	TAGTGGTGGTCGGAGATTCGTA
MRC1	ACCTGCGACAGTAAACGAGG	GGTGGGTTACTCCTTCTGCC
NOS2	CTCTATGTTTGCGGGGATGT	TTCTTCGCCTCGTAAGGAAA

### Statistics

To analyze differences in cell surface sFLT1, log-transformed geometric mean fluorescence intensities (MFI) were compared using two-way analysis of variance, linear mixed models or linear regression, as indicated in the figure legends. Where indicated, the intercept random effects of the independent experiments were included in the linear mixed models, to account for inter-experiment variance. For comparison of gene expression, normalized gene expression data were log-transformed and compared using Students’ t-test.

## Supplementary Information

Below is the link to the electronic supplementary material.

Supplementary file1 (DOCX 12 kb)

## Data Availability

The mass spectrometry and sequencing data supporting the findings of this study are openly available in repositories PRIDE and GEO.
