# Supplementary material for: The VEGF decoy receptor soluble Fms-like tyrosine kinase 1 binds to macrophages
Source: Angiogenesis. 2025 May 2;28(3):28. doi: 10.1007/s10456-025-09980-w (PMC12048422; doi:10.1007/s10456-025-09980-w)
Supplement: Supplementary file 1 — Supplementary file1 (DOCX 12 kb) [file 10456_2025_9980_MOESM1_ESM.docx]

**Supplemental file**

|  | *TNFA* | *IL6* | *IL1B* | *IL10* | *MRC1* |
| --- | --- | --- | --- | --- | --- |
| IFNg+LPS | 41.87 | 375.20 | 22.84 | 0.77 | 0.00 |
| IL-4 | 2.79 | 0.97 | 0.86 | 5.98 | 5.63 |

**Supplemental Table 1.** Differential activation of THP-1 macrophages with IFN-γ (20ng/ml) + LPS (1ng/ml) for 24h, or with IL-4 (10ng/ml, 48h) leads to the upregulation of macrophage markers. Values represent fold changes in mRNA expression compared to the respective BSA-incubated control samples (24h or 48h).
